# Efficacy and safety of lebrikizumab for the treatment of moderate-to-severe atopic dermatitis: a systematic review and meta-analysis

**DOI:** 10.3389/fphar.2024.1429709

**Published:** 2024-11-19

**Authors:** Jinger Lin, Min Luo, Qianwei Zhuo, Nuo Chen, Haosong Zhang, Yue Han

**Affiliations:** Department of Dermatology, The Union Hospital, Fujian Medical University, Fuzhou, China

**Keywords:** lebrikizumab, meta-analysis, atopic dermatitis, efficacy, safety

## Abstract

**Background:**

Lebrikizumab, an IL-13 immunomodulator, has shown recommendable effectiveness and safety in clinical studies for the treatment of moderate-to-severe atopic dermatitis (AD) in adolescents and adults.

**Objective:**

To evaluate the efficacy and safety of lebrikizumab in the treatment of moderate-to-severe AD through a meta-analysis.

**Methods:**

PubMed, Embase, Web of Science, Medline, and ClinicalTrials.gov databases were searched up to 8 August 2023. Randomized clinical trials of lebrikizumab treatment for moderate-to-severe AD were included by screening titles, abstracts, and papers.

**Results:**

Five studies involving 1,551 patients with AD were identified. Pooled analysis revealed significant improvements in the Eczema Area and Severity Index (EASI) score (SMD = −0.527; 95% CI = [−0.617, −0.436]), Investigator’s Global Assessment (IGA) score (RR = 2.122; 95% CI = [1.803, 2.496]), Body Surface Area (BSA) score (SMD = −0.608; 95% CI = [−1.099, −0.118]), SCORing Atopic Dermatitis (SCORAD) score (SMD = −0.441; 95% CI = [−0.633, −0.250]). Moreover, Pruritus Numeric Rating Scale (P-NRS) score, Patient-oriented Eczema Measure (POEM) scores, Sleep-loss score and Dermatology Life Quality Index (DLQI) scores showed similar results. Adverse events (AEs) (RR = 0.984; 95% CI = [0.907, 1.068]) for lebrikizumab showed no statistically significant difference compared to placebo, with similar results for serious adverse events (SAEs) (RR = 0.748; 95% CI = [0.410, 1.364]).

**Conclusion:**

This meta-analysis reveals that lebrikizumab has higher efficacy and safety in the treatment of moderate-to-severe AD, with the 250 mg Q2W dosage regimen appearing to be more advantageous.

## 1 Introduction

Atopic dermatitis (AD) is a chronic, recurrent, inflammatory skin disease characterized by dryness, itching, and eczema (Ali et al., 2020). The overall prevalence of AD is 15%–20% in children and up to 10% in adults. It may cause symptoms such as insomnia and anxiety affecting patients’ daily lives, reducing productivity, and increasing financial burden due to its recurrence (Sroka-Tomaszewska and Trzeciak, 2021; Schuler et al., 2023). The management of AD is tailored to the individual and depends on the severity of the condition. In addition to the use of general moisturizing care, emollients, and topical corticosteroids (TCSs) for the treatment of mild AD, topical calcineurin inhibitors (TCIs), crisaborole, phototherapy, immunosuppressants, and small molecule biological therapies may be used for the treatment of moderate-to-severe AD. The pathogenetic pathways of AD can be targeted by small molecule biological therapies with fewer negative effects (Fishbein et al., 2020; Sedeh et al., 2022).

As part of the TH2 immune system, interleukin-13 (IL-13) mediates AD inflammation. IL-13 and IL-4 share the same receptor subunit, the IL-4Rα chain, and IL-4Rα/IL-13Rα1 activates STAT6 via the JAK-STAT signaling cascade or tyrosine kinase 2, which in turn drives skin inflammation (Bieber, 2020; Newsom et al., 2020). An experiment observed that a reduction in IL-13/IL-4Rα resulted in a significant decline in scratching behavior, indicating that IL-13/IL-4Rα plays an important role in AD pathogenesis (Loh et al., 2020; Oetjen et al., 2017). Lebrikizumab, a fully human monoclonal antibody targeting IL-13, binds to IL-13 and prevents the dimerization of IL-13Rα1 and IL-4Rα (Labib et al., 2022).

Several recent clinical studies on lebrikizumab treatment have shown good efficacy and safety, but the results are not sufficiently reliable due to different sample sizes, doses, and intervals of administration (Wang et al., 2018). Therefore, we pooled data from different studies in a meta-analysis to analyze the efficacy and safety of lebrikizumab in the treatment of moderate-to-severe AD and attempted to identify better dosages and intervals of administration.

## 2 Methods

### 2.1 Search strategy

We conducted a comprehensive search for randomized control trials (RCTs, published up to 8 August 2023) in the PubMed, Embase, Web of Science, Medline, and ClinicalTrials.gov databases using the search terms “lebrikizumab” and “atopic dermatitis” or “eczema” or “dermatitis, atopic.” All articles retrieved using this strategy were independently checked by two authors (LJE and LM), and discrepancies were resolved through discussion and consensus. If discrepancies could not be resolved, a third-party assessment was used.

### 2.2 Inclusion and exclusion criteria

All RCTs were included based on the following criteria: 1) patients diagnosed with moderate-to-severe AD with at least a 1-year history who had been treated with lebrikizumab; 2) randomized, double-blind, placebo-controlled studies; and 3) the following outcomes were reported: Eczema Area and Severity Index (EASI), Investigator’s Global Assessment (IGA) score, pruritus score, Body Surface Area (BSA) score, SCORing Atopic Dermatitis (SCORAD) score, Pruritus Numerical Rating Scale (P-NRS) or Pruritus Visual Analog Scale (P-VAS) score, Patient-Oriented Eczema Measure (POEM) score, Sleep-loss score, and Dermatology Life Quality Index (DLQI) score. The criteria for defining moderate-to-severe AD were as follows: IGA scores of 3 or higher at screening and baseline, including moderate (IGA = 3) and severe (IGA = 4); EASI scores of 14 or higher at screening and 16 or higher at baseline; BSA affected by more than 10%; poor response to topical medication.

RCTs were excluded based on the following criteria: 1). non-English language articles; 2) unavailability of full text; 3). non-RCT trials; and 4) reviews, abstracts, case reports, meta-analyses, or guidelines.

### 2.3 Data extraction and outcome measures

The data extracted from these RCTs included study references, duration of the study, number of lebrikizumab-treated patients and placebo subjects, gender and age of the group, the dosage of lebrikizumab used, subject interventions, and outcome measures. Given that our aim was to explore the efficacy and safety of lebrikizumab in the treatment of AD patients, the efficacy outcomes included EASI, IGA, BSA, SCORAD, P-NRS or P-VAS, POEM, Sleep-loss score, and DLQI, and the safety outcomes comprised adverse events (AEs) and serious adverse events (SAEs).

### 2.4 Risk assessment of bias

An independent quality assessment of the included studies was conducted by two researchers (LJE and LM) according to the Cochrane Handbook of Systematic Reviews 5.1.0, and the results were cross-checked. The evaluation included a randomization process, deviation from the intended intervention, missing outcome data, outcome measures, and selection reporting. The judgments for each assessment were graded on three levels: high (high risk of bias), low (low risk of bias), and some (medium risk of bias) concerns. Funnel plotting was used to detect the bias of publications (Li et al., 2023).

### 2.5 Statistical analysis

Forest plots were performed by STATA 12.0. Data were presented as standardized mean difference (SMD) or relative risk (RR). Heterogeneity was calculated using I^2^ statistics and chi-squared test. If the I^2^ value was greater than 50%, heterogeneity was considered high, and a random-effects model was used. A *p*-value of <0.05 was considered statistically significant. A Galbraith radial plot was conducted to indicate the cause of heterogeneity.

## 3 Results

### 3.1 Search results

**FIGURE 1:**
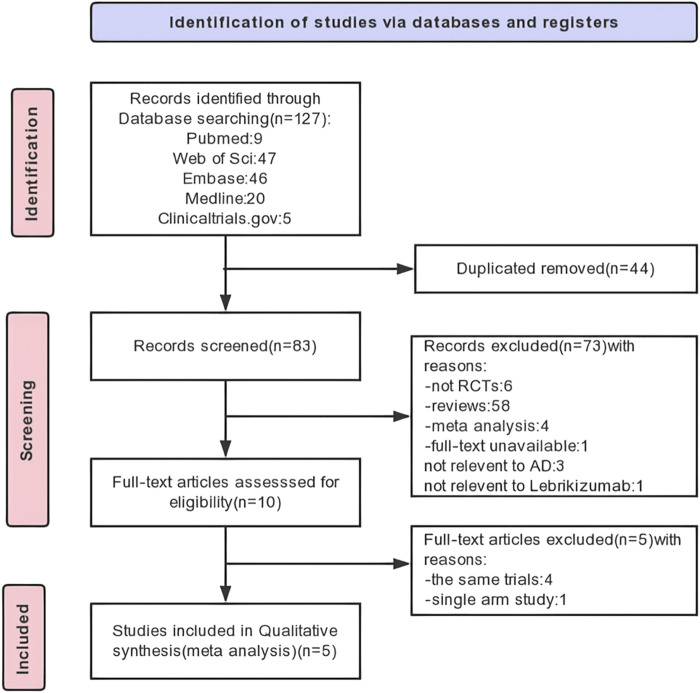
PRISMA flow diagram of study selection.

We identified 122 original articles, and five RCTs were enrolled. A total of 44 duplicates and 73 articles not meeting the requirements were excluded after record screening. After full-text screening, five RCTs were finally selected for analysis, of which four identical trials and one single-arm trial were excluded. The detailed process and results of article screening are shown in Figure 1.

### 3.2 Study characterization

Of the 1,551 participants included in the studies, 784 were male and 767 were female. Three RCTs (Silverberg et al., 2023; Blauvelt et al., 2023; Simpson et al., 2023) included adolescents as subjects, defined as those aged 12–17 years and weighing ≥40 kg (Simpson et al., 2023; Silverberg et al., 2023; Blauvelt et al., 2023). All studies allowed long-term use of topical medications without study interruption. These trials were conducted in the United States and the European Union. The study population was 63.3% white and 19.2% Asian individuals (Supplementary Table S1). Parallel phase III trials with identically constructed research (Silverberg et al., 2023; Blauvelt et al., 2023) were recorded during two time periods. The first phase was an induction period, which lasted from baseline to week 16 and consisted of injections of 500 mg of lebrikizumab as a loading dose (LD) at baseline and week 2 (Silverberg et al., 2023). This was followed by a single injection given fortnightly from weeks 4–14 for a total of 16 weeks of observation, and participants were re-randomized to receive a placebo, lebrikizumab 250 mg Q2W, or lebrikizumab 250 mg Q4W. The second phase was a maintenance period lasting 36 weeks from weeks 16–52 (Blauvelt et al., 2023). Considering that Blauvelt’s trial population was from a combination of the maintenance primary population (MPP) for ADvocate1 and the modified maintenance primary population (mMPP) for ADvocate2, this trial population was not double-counted when calculating the total sample, male, female, and ethnicity data. The characteristics of the five studies are shown in Table 1 (Guttman-Yassky et al., 2020; Simpson et al., 2018).

**TABLE 1:** Characteristics of the included RCTs in the meta-analysis.

Reference (study, year, phase)	Treatment wks	Study design	Patients (Leb/PBO)	Sex (female), n (%)	Age, mean ± SD, y	Dosage, mg	Outcome measure
Silverberg (ADvocate1) 2023, III	16	RCT DB PC	424 (283/141)	214 (50.5)	35.5 ± 17.3	500 LD at baseline and week 2, followed by 250 Q2W	①②③④⑤⑦⑧⑨
Silverberg (ADvocate2) 2023, III	16	RCT DB PC	427 (281/146)	211 (49.4)	36.2 ± 16.9	500 LD at baseline and week 2, followed by 250 Q2W	①②③④⑤⑦⑧⑨
Blauvelt (ADvocate1) 2023, III	52	RCT DB PC	157 (125/32)^†^	87 (55.4)	35.1 ± 17.5	250 Q2W and 250 Q4W^a^	①②④⑤⑧⑨
Blauvelt (ADvocate2) 2023, III	52	RCT DB PC	134 (106/28)^‡^	71 (53.0)	35.8 ± 16.8	250 Q2W and 250 Q4W^b^	①②④⑤⑧⑨
Simpson, 2023, III (ADhere)	16	RCT DB PC	211 (145/66)	103 (48.8)	37.4 ± 19.3	500 LD at baseline and week 2, followed by 250 Q2W	①②③④⑤⑦⑧⑨
SIimpson, 2018, II (TREBLE)	12	RCT DB PC	209 (156/53)	73 (34.9)	36.15 ± 12.7	125 and 250 single doses at baseline, 125 Q4W	①②③④⑥⑦⑧⑨
Guttman-Yassky, 2020, IIb	16	RCT DB PC	280 (228/52)	166 (59.3)	39.3 ± 17.5	125 Q4W (250 LD at baseline), 250 Q4W (500 LD at baseline), and 250 Q2W (500 LD at baseline and week 2)	①②③⑤⑦⑧⑨

Leb, lebrikizumab; PBO, placebo; RCT, randomized control trial; DB, double-blind; PC, placebo-controlled; mg, milligram; wks, weeks; SD, standard deviation; Q2W, every 2 weeks; Q4W, every 4 weeks; LD, loading dose. Outcome measures: ①EASI, Eczema Area and Severity Index; ②IGA, Investigator’s Global Assessment; ③BSA, Body-Surface Area; ④SCORAD, SCORing Atopic Dermatitis; ⑤P-NRS, Pruritus Numerical Rating Scale; ⑥P-VAS, Pruritus Visual Analogue Scale; ⑦ POEM, Patient-Oriented Eczema Measure; ⑧Sleep-loss score; ⑨DLQI, Dermatology Life Quality Index; a and b, dosing regimen follows Silverberg’s trials where participants were re-randomized to dosing after week 16. †, maintenance primary population, ‡, modified maintenance primary population.

### 3.3 Risk of bias and quality assessment

**FIGURE 2:**
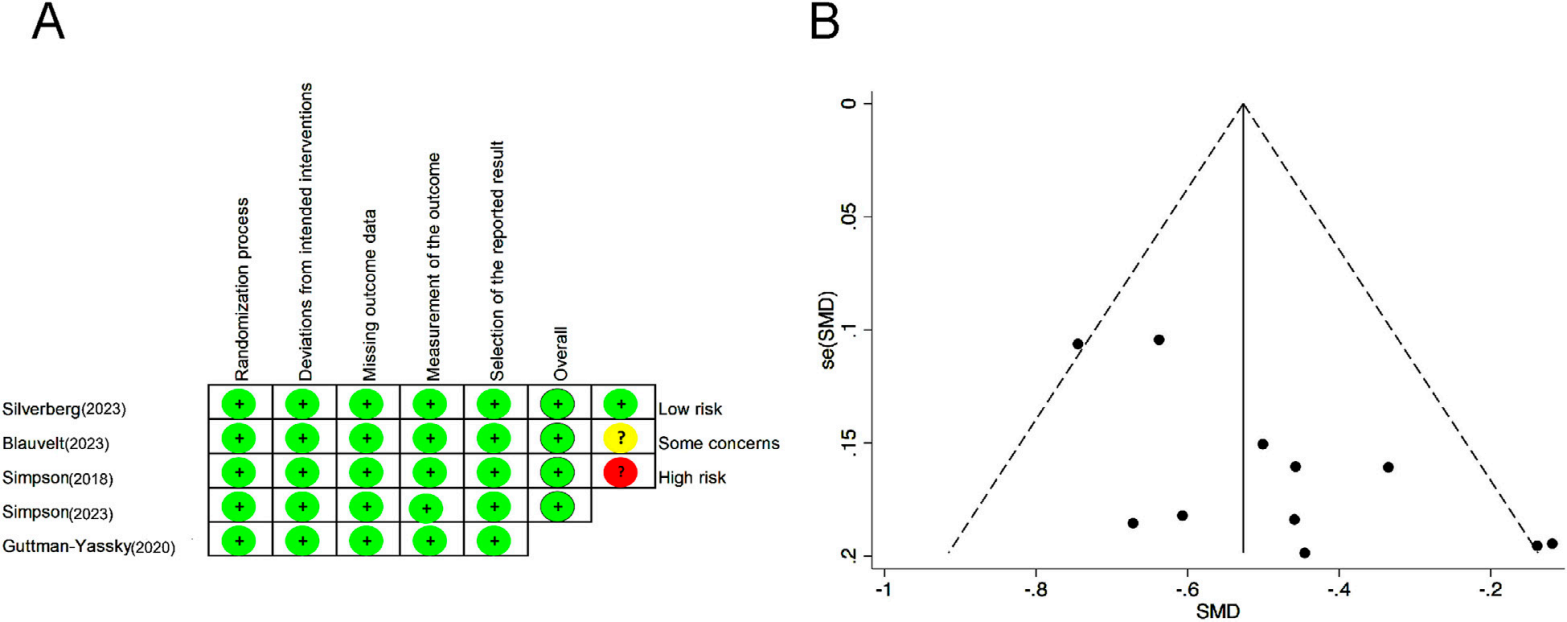
Risk of bias summary for each included RCT. **(A)** Bias risk assessment according to the Cochrane Handbook of Systematic Reviews 5.1.0. **(B)** Funnel plot to detect publication bias. RCTs, randomized control trials.

The risk of bias and quality assessment were evaluated by ROB2 and the Jadad scale, respectively. All studies had clear and adequate random methods, allocation concealment, complete blinding of participants and personnel, complete outcome data, and the option to report results, leading to a low risk of bias; see Figure 2A for the detailed bias risk assessment. The Jadad scale showed the high study quality of each RCT (Supplementary Table S2). Upon meticulous examination of the funnel plot, it was clear that the articles incorporated in this meta-analysis exhibited relatively modest publication bias (Figure 2B).

### 3.4 Efficacy

There is no single outcome measure that can comprehensively assess the severity of AD, and a complete evaluation of the efficacy of AD can be made in terms of signs, symptoms, and quality of life (Narla and Silverberg, 2023). The following scores were used to evaluate efficacy: EASI, SCORAD, IGA, BSA, P-NRS or P-VAS, Sleep-loss score, DLQI, and POEM. The greater the reduction in endpoint scores compared to baseline, the stronger the evidence to believe that lebrikizumab is effective in treating moderate-to-severe AD. In conclusion, lebrikizumab was significantly more efficient than placebo.

**FIGURE 3:**
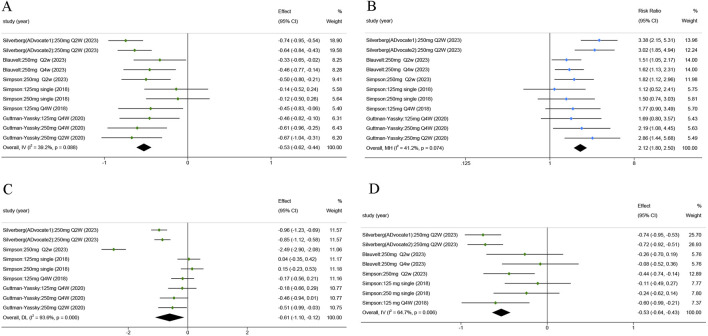
Forest plots of the efficacy of lebrikizumab treatment in the 5 RCTs: **(A)** Eczema Area and Severity Index (EASI) score; **(B)** Investigator’s Global Assessment (IGA) score; **(C)** Body Surface Area (BSA) score; **(D)** SCORing Atopic Dermatitis (SCORAD) score. Horizontal lines represent 95% CIs of the RR estimates. Green dots represent the SMD, blue dots represent RR, and diamonds represent the meta-analysis summary effect estimate. RCTs, randomized control trials; RR, relative risk; SMD, standardized mean difference.

Lebrikizumab groups showed a substantial decline in the EASI score throughout all trials (SMD = −0.527; 95% CI = [−0.617, −0.436]; z = −11.403; *p* < 0.001; Figure 3A). Five RCTs reported the IGA (0/1), and the pooled analysis indicated that the lebrikizumab 250 mg Q2W group had significantly better score reduction (RR = 2.122; 95% CI = [1.803, 2.496]; z = 9.064; *p* < 0.001; Figure 3B). Higher BSA scores corresponded to a higher burden of AD despite mild lesions (Narla and Silverberg, 2023). The SMD was −0.608 in the group treated with lebrikizumab (95% CI = [−1.099, −0.118]; z = −2.431; *p* < 0.05; Figure 3C). SCORAD evaluates the extent of AD lesions and can be used as an indicator of both signs and symptoms. In the included studies, the SMD of the SCORAD score in the lebrikizumab-treated groups was −0.441(95% CI = [−0.718, −0.312]; z = −4.966; *p* < 0.001, Figure 3D).

**FIGURE 4:**
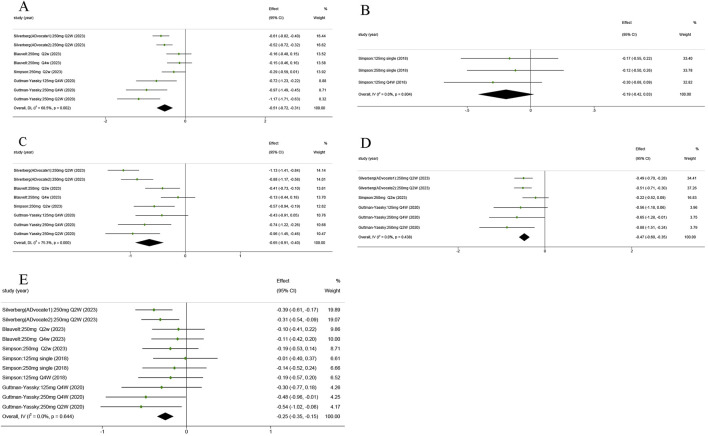
Forest plots of the efficacy of lebrikizumab treatment in the five RCTs: **(A)** Pruritus Numerical Rating Scale (P-NRS) score; **(B)** Pruritus Numerical Rating Scale (P-VAS) score; **(C)** Patient-Oriented Eczema Measure (POEM) score; **(D)** Sleep-loss score; **(E)** Dermatology Life Quality Index (DLQI) score. Horizontal lines represent 95% CIs of the RR estimates. Green dots represent the SMD, and diamonds represent the meta-analysis summary effect estimate. RCTs, randomized control trials; RR, relative risk; SMD, standardized mean difference.

AD patients always experience a vicious cycle of itch–scratch–itch (Bawany et al., 2021). There was a significant improvement in P-NRS in the lebrikizumab-treated group compared to the placebo group (SMD = −0.515; 95% CI = [−0.718, −0.312]; z = −4.966; *p* < 0.01; Figure 4A). No significant statistical difference was observed when comparing the P-VAS of the placebo group (SMD = −0.194; 95% CI = [−0.415, 0.027]; z = −1.717; *p* = 0.086; Figure 4B). POEM is a viable method for assessing AD symptoms (Silverberg et al., 2018). Pooled analysis showed a quantifiable improvement in lebrikizumab-treated groups (SMD = −0.653; 95% CI = [−0.911, −0.395]; z = −4.961; *p* < 0.001; Figure 4C). AD in 47%–80% of children and 33%–90% of adults was associated with sleep disturbance. Sleep-loss score in the lebrikizumab-treated groups revealed considerable improvement compared to placebo groups (SMD = −0.475; 95% CI = [−0.598, −0.351]; z = −7.513; *p* < 0.001; Figure 4D).

The global Harmonizing Outcome Measures for Eczema (HOME) recommended DLQI as the preferred measure of quality of life in adults (age ≥18 years) (Narla and Silverberg, 2023). All RCTs reported that the DLQI of the 250 mg Q2W lebrikizumab groups exceeded that of the matched placebo groups (SMD = −2.52; 95% CI = [−0.350, −0.153]; z = −5.016; *p* < 0.001; Figure 4E).

### 3.5 Safety

**FIGURE 5:**
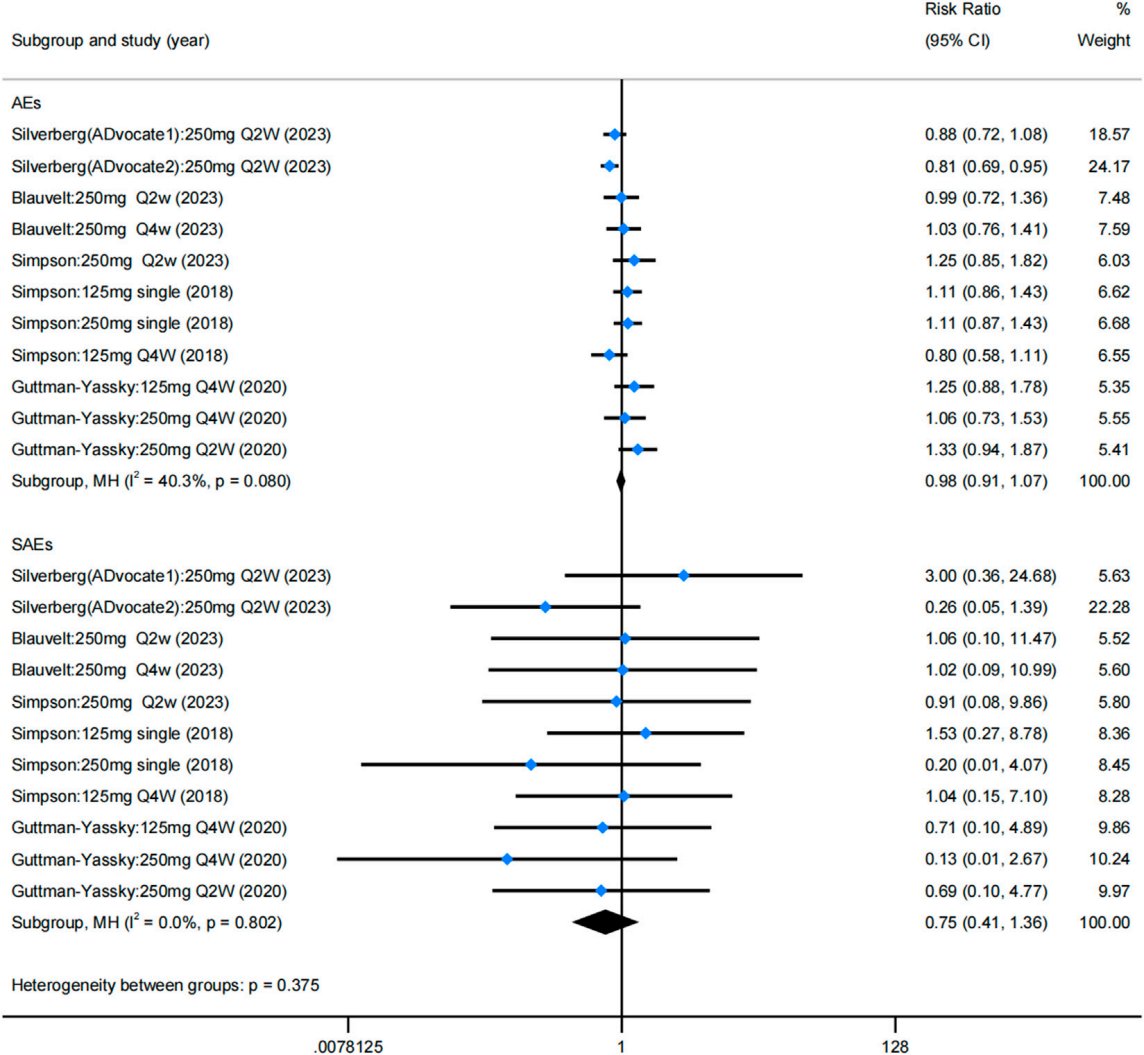
Forest plots of rate of AEs and SAEs in the five RCTs. Blue dots represent the RR, and diamonds represent the meta-analysis summary effect estimate. RCTs, randomized control trials; RR, relative risk; AEs, adverse events; SAEs, serious adverse events.

These results revealed that lebrikizumab has an acceptable safety profile, with no statistically significant difference in AEs and SAEs compared with the placebo group (Figure 5). The rate of AEs among different trials showed no statistical significance (RR = 0.984; 95% CI = [0.907, 1.068]; z = −0.384; *p* = 0.701), and the rate of SAEs was also not significantly different between the placebo and lebrikizumab groups (RR = 0.748; 95% CI = [0.410, 1.364]; z = 9.064; *p* = 0.343). Combining these results, the overall incidence of AEs was 50%, and SAEs accounted for 2% (Supplementary Table S3). Common AEs included infections (10.9%), conjunctivitis (9.4%), nasopharyngitis (6.3%), headache (4.2%), herpes infection (4.0%), skin infections (3.6%), injection site reactions (2.5%), dry eye (2.0%), potential opportunistic infections (1.1%), and eosinophilia (1.1%). SAEs manifested primarily as isolated events, and no deaths were documented as a result of lebrikizumab use.

A few cases of malignancy were reported in separate trials conducted by Silverberg et al. and Blauvelt et al., respectively. There was one case of T-cell lymphoma, one case of pancreatic cancer characterized by bone and liver metastases, and one case of ovarian germ cell teratoma. However, no cases of melanoma were detected during these trials.

### 3.6 Heterogeneity test

**FIGURE 6:**
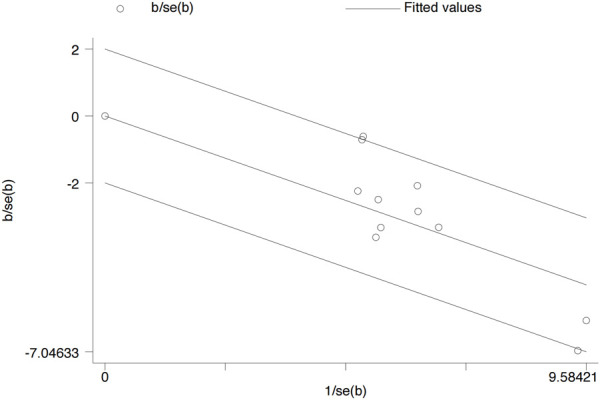
Galbraith plot for each included RCT. RCTs, randomized control trials.

A Galbraith radial plot (Figure 6) was performed to identify the major source of the heterogeneity of overall effects, indicating that IGA (I^2^ = 41.2% and *p* = 0.074), BSA (I^2^ = 9 3.6% and *p* < 0.001), SCORAD (I^2^ = 64.7% and *p* = 0.006), P-NRS (I^2^ = 68.5% and *p* = 0.002), and POEM (I^2^ = 75.3% and *p* < 0.001) may significantly affect the heterogeneity of the outcomes; meanwhile, EASI (I^2^ = 39.2% and *p* = 0.088), P-VAS (I^2^ = 0.0% and *p* = 0.804), Sleep-loss score (I^2^ = 0.0% and *p* = 0.438), DLQI (I^2^ = 0.0% and *p* = 0.644), AEs (I^2^ = 40.3% and *p* = 0.080), and SAEs (I^2^ = 0.0% and *p* = 0.802) were not observed to be heterogeneous between the lebrikizumab-treated and placebo groups.

## 4 Discussion

Our meta-analysis shows that the efficacy of lebrikizumab is superior to that of placebo based on comparisons of different outcome measures, and there were no deaths related to lebrikizumab treatment. Long-term (52-week) efficacy was observed in only one of these RCTs (Blauvelt et al., 2023), while the remaining trials had treatment durations of 12 or 16 weeks.

In the previous systematic review, a pooled analysis including three RCTs was conducted, examining the efficacy of lebrikizumab as assessed by available data on EASI-75, IGA, and BSA scores (Bashrahil et al., 2022). However, this current meta-analysis expands upon prior work by incorporating two additional RCTs and investigating the long-term effects of lebrikizumab treatment. The analysis was expanded to include assessments of the SCORAD, P-NRS, P-VAS, POEM, and Sleep-loss scores. Notably, these were designed to provide a more comprehensive understanding of the therapeutic potential of lebrikizumab. Consistent with the previous studies, analysis of the RCTs reaffirms the favorable efficacy profile of lebrikizumab.

A forest plot of EASI scores showed the highest efficacy in the 250 mg Q2W and 250 mg Q4W groups. Similarly, promising outcomes were observed for the IGA scores in most groups; however, no significant improvement was found in the 125 mg Q4W group at weeks 12 and 16 (Simpson et al., 2018; Guttman-Yassky et al., 2020). In contrast, the SCORAD scores exhibit a nuanced response. Although more favorable outcomes were observed at the 12- and 16-week follow-ups, the decrease in SCORAD was not significant at week 52 (Blauvelt et al., 2023). This observation highlights the challenging nature of reducing SCORAD scores, suggesting its potential limitations as an indicator in extended trials. Similarly, the performance of P-NRS showed an improving trend of progressive reduction over time, particularly evident at 12, 16, and 52 weeks. POEM analysis revealed that the 250 mg Q2W regimen was superior to the 250 mg Q4W regimen, with the 125 mg Q4W regimen being the least effective. Consequently, we concluded that the best results were achieved by taking 250 mg every fortnight. This trend was further reflected in the results of sleep loss VRS and DLQI assessments, with the 250 mg Q2W group consistently demonstrating the most favorable outcomes.

In summary, single-doses of 125 mg and 250 mg failed to significantly improve most endpoints in moderate-to-severe AD. Thus, their efficacy in the treatment of moderate-to-severe AD is questionable. In contrast, the 250 mg Q2W regimen consistently displays the most promising performance across both short- and long-term trials.

Allergic comorbidities (e.g., conjunctivitis and nasopharyngitis) and injection site reactions are common AEs to lebrikizumab. Similarly, IL-13 inhibitors used to treat moderate-to-severe AD, including dupilumab, cendakimab, and eblasakimab, have been associated with these common side effects. A retrospective study involving 165 adult AD patients treated with dupilumab showed rhinitis (21.8%) and conjunctivitis (10.9%) (Napolitano et al., 2021). The incidence of injection site reactions during dupilumab treatment for AD can be as high as 28% (Patruno et al., 2022). In a Phase II RCT of cendakimab (NCT04800315), ocular allergic comorbidities occurred in 11.6%, nasopharyngeal allergic symptoms in 5.9%, and injection site reactions in 6.1% of patients (A et al., 2024). In an 8-week Phase Ib RCT of eblasakimab, the incidence of allergies (e.g., dust, pets, and seasonal allergies) was 37%, and 26% of patients reported injection site reactions (Veverka et al., 2024). In a pooled analysis of five Phase II and III RCTs involving tralokinumab for moderate-to-severe AD, it was found that allergies (e.g., seasonal allergies, food, animals, and mites) accounted for 65.8% of cases, with specific categories including allergic rhinitis for 9.5%, and allergic conjunctivitis for 19.8% (Simpson et al., 2022). This highlights the need to monitor similar AEs across biologics with the same antagonistic mechanism to investigate whether these reactions are associated with IL-13 blockade in future studies.

Unfortunately, although some of the study subjects were adolescents, their results were not reported separately. However, ADore (NCT04250350), a single-arm trial that was excluded from our initial rigorous screening, continues to play an important role in analyzing the long-term treatment with lebrikizumab in adolescents. This was a 52-week Phase 3, open-label study in which adolescents with moderate-to-severe AD received subcutaneous lebrikizumab (500 mg LD at baseline and week 2, followed by 250 mg Q2W thereafter). At week 52, 62.6% of treated adolescents achieved an IGA score of 0 or 1, and 81.9% achieved EASI-75. Significant improvements were also observed in DLQI/CDLQI, PROMIS Anxiety, and PROMIS Depression scores (Paller et al., 2023). Common AEs included nasopharyngitis (9.7%), conjunctivitis (6.8%), and injection site reactions (2.4%). These were consistent with our previous observations.

## 5 Limitations

The validity of the results may be compromised by the limited sample size and lack of racial diversity in the pooled studies as people of different races and pigmented skin have different immunological profiles (Bernardo et al., 2023; Arents et al., 2023). The criteria used to define moderate-to-severe AD varied from study to study; for example, Simpson et al. (2023) used the American Academy of Dermatology Consensus Criteria, whereas Guttman-Yassky et al. (2020) used the Hanifin and Rajka’s criteria. The use of TCSs to maintain patient compliance may also influence the final outcomes of lebrikizumab. Furthermore, not all trials included adolescents, and most were short-term observations, which may have led to a poor understanding of the potential complications of long-term lebrikizumab treatment and its effects on adolescents. The trial conducted by Simpson et al. (2018) did not administer a baseline LD, and it is unknown whether this would have made a difference compared to trials that administered a LD. As more RCTs on lebrikizumab emerge, we expect that its clinical efficacy and safety will be analyzed from multiple perspectives. Additional databases will be considered for future analyses of lebrikizumab.

## 6 Conclusion

Skin lesions in AD patients exhibit T-cell infiltration, and the primary pathogenesis is the strong activation of type 2 immune responses driven by Th2 cells and their signature cytokines IL-4 and IL-13 (Langan et al., 2020). Lebrikizumab, an IL-13 bioinhibitor, has demonstrated excellent efficacy and safety in several Phase II and III RCTs. Our systemic review aims to provide clinicians with more evidence-based medical information to choose lebrikizumab in the treatment of moderate-to-severe AD, leading to better results in combination therapy.

## Data Availability

The original contributions presented in the study are included in the article/Supplementary Material; further inquiries can be directed to the corresponding authors.

## Abbreviations

AD, atopic dermatitis; IL-13, interleukin-13; LD, loading dose; Q2W, every 2 week; Q4W, every 4 week; RCTs, randomized control trials; EASI, eczema area and severity index; IGA, investigator’s global assessment; BSA, body surface area; SCORAD, scoring atopic dermatitis; P-NRS, pruritus numerical rating scale; P-VAS, pruritus visual analog scale; POEM, patient-oriented eczema measure; DLQI, dermatology life quality index; SMD, standardized mean difference; AEs, adverse events; SAEs, serious adverse events; RR, relative risk.
